# Association between body fat distribution and age at menarche: a two sample Mendelian randomization study

**DOI:** 10.3389/fped.2024.1349670

**Published:** 2024-04-08

**Authors:** Peng Xue, Dan Wang, Yao Chen, Jingyi Tang, Yang Chen, Hao Mei, Cuilan Lin, Shijian Liu

**Affiliations:** ^1^ Hainan Branch, Shanghai Children's Medical Center, School of Medicine, Shanghai Jiao Tong University, Sanya, China; ^2^ School of Public Health, Shanghai Jiao Tong University, Shanghai, China; ^3^ Pediatric Translational Medicine Institute, Shanghai Children's Medical Center, School of Medicine, Shanghai Jiao Tong University, Shanghai, China; ^4^ Department of Endocrinology and Genetic Metabolism, Shanghai Children's Medical Center, School of Medicine, Shanghai Jiao Tong University, Shanghai, China; ^5^ Department of Data Science, University of Mississippi Medical Center, Jackson, MS, United States; ^6^ Boai Hospital of Zhongshan, South Medical University, Guangdong, China

**Keywords:** age at menarche, fat percentage, fat mass, MR Bayesian model averaging, Mendelian randomization

## Abstract

**Background:**

Numerous studies have examined the association between obesity and age at menarche (AAM), with most focusing on traditional obesity indicators such as body mass index. However, there are limited studies that explored the connection between body fat distribution and AAM, as well as a scarcity of Mendelian randomization (MR) studies.

**Methods:**

In this study, we conducted a two-sample MR study to evaluate the causal effects of eight body fat distribution indicators on AAM. Inverse variance weighted (IVW) method was used for primary analysis, while supplementary approaches such as MR-Egger and weighted median were also utilized. Considering that the eight exposures were highly correlated, we performed an MR Bayesian model averaging (MR-BMA) analysis to prioritize the effect of major exposure on AAM. A series of sensitivity analyses were also performed.

**Results:**

From a range of 82–105 single nucleotide polymorphisms (SNPs) were utilized as genetic instrumental variables for each of the exposure factors. After Bonferroni correction, we found that whole body fat mass (*β*: −0.17; 95% CI: −0.24, −0.11), left leg fat percentage (*β*: −0.14; 95% CI: −0.21, −0.07), left leg fat mass (*β*: −0.20; 95% CI: −0.27, −0.12), left arm fat percentage (*β*: −0.18; 95% CI: −0.26, −0.11) and left arm fat mass (*β*: −0.18; 95%CI: −0.26, −0.10) were associated with decreased AAM using random effects IVW method. And the beta coefficients for all MR evaluation methods exhibited consistent trends. MR-BMA method validated that left arm fat percentage plays a dominant role in AAM.

**Conclusions:**

Our MR study suggested that body fat has broad impacts on AAM. Obtaining more information on body measurements would greatly enhance our comprehension of pubertal development.

## 1 Introduction

Over the last several decades, the weight problems, overweight and obesity, have been increasing constantly in Europe (1), with the obesity prevalence rates of ∼20% (2). And childhood obesity also cannot be ignored, with around 30% of children overweight or obese in Europe (3). At the same time, the past century has seen a dramatic decline in the age of menarche among European women (4). Epidemiological studies have consistently demonstrated a link between overweight or obesity and pubertal development (5, 6). These studies have shown that children who are overweight or obese tend to experience earlier onset of puberty, while those who are underweight or have a low body mass index (BMI) often have delayed pubertal timing, especially among girls (7). This correlation may be due to a variety of factors, including hormonal imbalances, genetics, lifestyles such as diet and physical activity, and others (8). Additionally, the timing of puberty has important implications for long-term health outcomes, with early puberty associated with increased risk of certain health conditions such as breast cancer and cardiovascular disease (9–11). Therefore, understanding the relationship between body composition and puberty is important for promoting health and preventing disease across the lifespan.

There are many phenotypes related to obesity, with the most commonly used one being BMI. However, BMI alone does not provide enough specific information and cannot reflect the distribution of body fat. So, previous studies using BMI to indicate obesity cannot effectively reflect the influence of body fat on children's pubertal development. Several observational epidemiological studies have attempted to investigate the relationship between body composition and breast development or age of menarche in girls (12–16). The study revealed a correlation between body fat percentage and fat mass with the onset of thelarche or menarche in girls. However, these investigations lacked the inclusion of fat distribution indicators in specific body regions, such as the arm or leg fat percentage. Furthermore, it is crucial to emphasize that conventional observational studies frequently encounter inherent limitations, such as residual confounding and reverse causality, which pose challenges in identifying consistent factors influencing pubertal development.

The Mendelian randomization (MR) design, which is a recently developed research approach, has been widely employed to examine the causal relationship between a particular exposure and a specific outcome (17). As an analytic approach, MR design provides a distinct advantage in assessing causality compared with observational studies by utilizing genetic variants as instrumental variables. By using genetic variants, which are randomly allocated at conception and are not influenced by any environmental or lifestyle factors, the MR design can help overcome confounding and reverse causality biases that may arise in traditional observational studies (18).

Currently, there is a paucity of research investigating the association between body fat distribution and pubertal development in the European population. Furthermore, the absence of MR study hinders a comprehensive understanding of the causal link between body fat distribution and the initiation of puberty in children. To bridge this gap, we undertook a two-sample MR study utilizing data from the UK Biobank. This study aims to elucidate the causal association between age at menarche (AAM) and body fat distribution, while also identifying most influential body fat distribution indicator on AAM.

## 2 Methods

### 2.1 Study design

**Figure 1:**
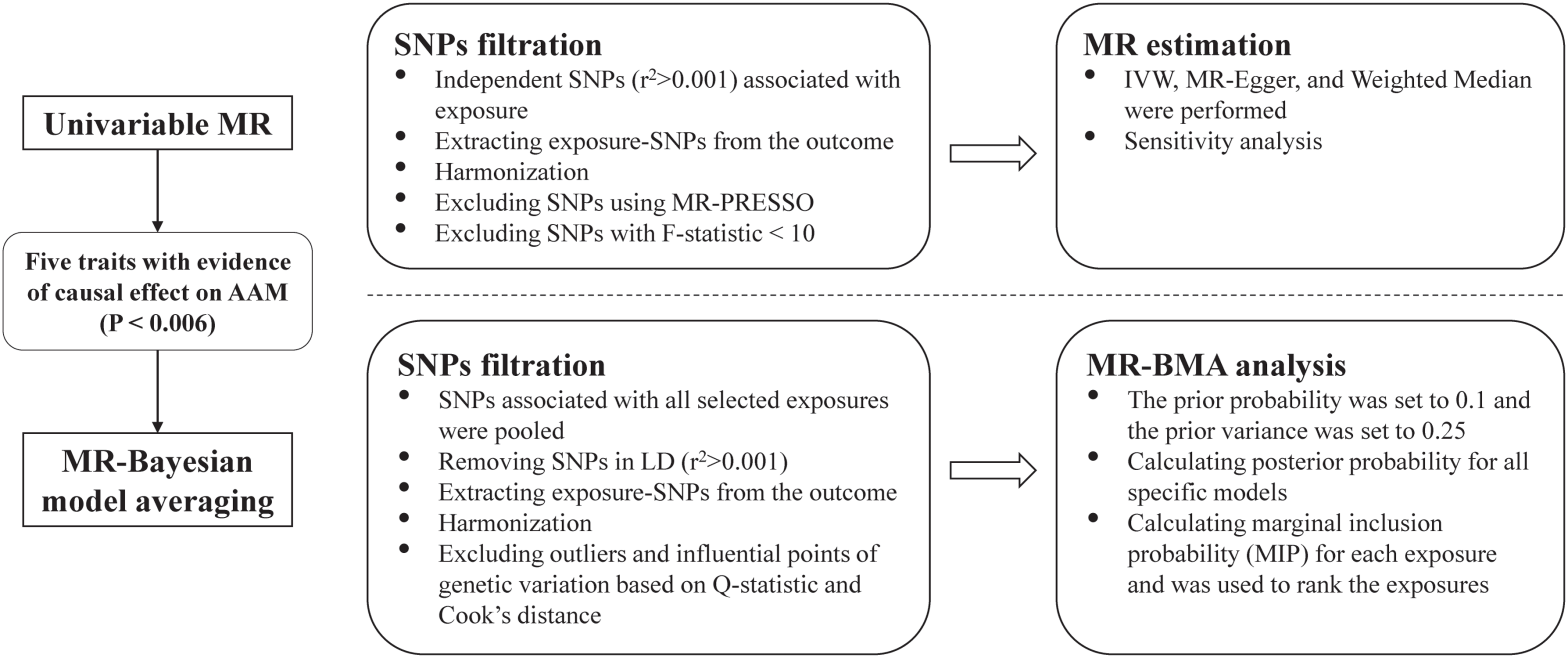
Overview of the study. SNP, single nucleotide polymorphism; MR-PRESSO, Mendelian randomization-pleiotropy residual sum and outlier; MR, Mendelian randomization; IVW, inverse-variance weighted, MR-BMA, MR Bayesian model averaging; LD, linkage disequilibrium.

We conducted a standard two-sample MR study to explore the causality between several anthropometric traits related to body fat distribution and the AAM. Firstly, we used univariable MR methods to explore the potential causal relationship between the eight exposures related to body fat distribution and AAM. Considering that the eight exposures are highly correlated, MR Bayesian model averaging (MR-BMA) method (19) was further used to prioritize the body fat distribution traits in order to identify the predominant trait associated with AAM. The study design is shown in Figure 1. Statistical analysis was performed by the “TwoSampleMR” (Version 0.5.6) and “mrbma” package of the R program (Version 4.2.0). Our study primarily relied on summary-level statistics and thus did not require ethical approval.

### 2.2 Data sources

Summary-level data of exposure were extracted from the second round of Neale Lab's GWAS (http://www.nealelab.is/uk-biobank) in UK Biobank. Researchers measured body composition via bioelectrical impedance analysis using a Tanita BC418MA body composition analyzer, gaining many anthropometric measures, such as whole-body fat mass, whole-body fat-free mass, fat mass, and nonfat mass for each of trunk, arm, and leg anthropometric traits. Among them, we included a total of 8 variables related to fat distribution, including body fat percentage, whole body fat mass, left leg fat percentage, left leg fat mass, left arm fat percentage, left arm fat mass, trunk fat percentage, and trunk fat mass. Furthermore, we solely utilized data coming exclusively from women, which included approximately 191,000 samples.

Genetic association estimates with the outcome were extracted from the largest meta-genome-wide association studies (GWAS) of AAM incorporating 252,514 women collected by the ReproGen consortium (*N* = 179,117) and UK Biobank (*N* = 73,397) (20).

### 2.3 Selection of genetic instruments for body fat distribution

As independent genetic instrumental variables (IVs), single nucleotide polymorphisms (SNPs) were associated with the appropriate exposure at the genome-wide significance threshold *P* < 5 × 10^−8^. Additionally, we clumped and discarded SNPs at the threshold of linkage disequilibrium (LD) with a threshold of *r*^2^ > 0.001 within a 10,000-kilobase window, using the 1,000 Genomes European reference panel. We then performed SNPs filtering according to a series of procedures: (a) removed the SNPs associated with outcomes at genome-wide significance; (b) harmonized the exposure-outcome datasets to exclude palindromic and incompatible SNPs with intermediate allele frequencies; (c) then implemented MR-pleiotropy residual sum and outlier (MR-PRESSO) to identify and remove SNPs with potential pleiotropy and outliers, with a threshold of *P* < 0.05. *F*-statistics were finally calculated to quantify the strength of the selected SNPs. When the corresponding *F*-statistic is >10, it is considered to be sufficient (21).

### 2.4 Univariable MR estimations

We used three MR approaches, namely the inverse-variance weighted (IVW), weighted median, and MR-Egger, to determine MR estimates of body fat distribution for AAM. The IVW method, operating under the random-effects model, was used for primary MR analysis, which provided the most precise estimates by assuming that all SNPs are valid instruments (22). MR-Egger and weighted median methods were used to complement IVW estimates as these approaches could provide more robust results. The weighted median model can offer a reliable estimation of causality when a minimum of 50% of IVs demonstrate effectiveness are effective (23). On the other hand, the MR-Egger regression allows for all instrumental variants to be invalid and provides a robust estimate, but it necessitates the fulfillment of the Instrument Strength Independent of Direct Effects (InSIDE) assumption by the variants (24). The consistency in the direction of estimates across all MR methods enhances confidence in the causal evidence. A statistically significant result was determined using a threshold of *P* < 0.006 (0.05 divided by 8 exposures). Nominal significant results were identified at *P* < 0.05 threshold.

### 2.5 MR Bayesian model averaging (MR-BMA)

For the exposure which presented a statistic significance on AAM after Bonferroni correction, we used the MR-BMA analysis to discover the body fat distribution trait that play predominant role in the causal associations with AAM. Compared with conventional multivariable MR methods, the MR-BMA method can detect true causal risk factors even when the candidate risk factors are highly correlated (19). SNPs associated with all selected exposures were pooled. Posterior probability (PP) was calculated for all specific models (i.e., one trait or a combination of multiple traits). The marginal inclusion probability (MIP) for each exposure, which is the sum of the PP over all models where the exposure is present, was used to rank the causal associations of the body fat distribution indicators with AAM. We also calculated model-averaged causal effects (MACE), which demonstrates the direct causal effect of a body fat distribution trait on AAM averaged across all related models. Notably, these estimates can be used to compare exposures or to interpret effect directions, but should not be interpreted absolutely.

As previously described (25), the prior probability was set to 0.1 and the prior variance was set to 0.25. A stochastic search with 10,000 iterations was undertaken and empirical *p* values with 10,000 permutations were calculated.

### 2.6 Pleiotropy assessment and sensitivity analysis

In the univariable MR analyses, to assess whether IVs affect the level of pleiotropic effects of outcomes through more than one biological pathway, we used MR-Egger regression to test for evidence of pleiotropy. A zero intercept from MR-Egger (*P* > 0.05) indicates no potential horizontal pleiotropy (24). For potential heterogeneity among causal effects of different variants, we used Cochran's *Q* statistic from IVW (together with the *I*^2^ statistic) to detect it (26). Cochran's *Q* test with *P *< 0.05 and *I*^2^ > 50% indicates the presence of potential heterogeneity. However, it is important to note that the presence of heterogeneity does not necessarily render the random-effects IVW estimate invalid when the overall heterogeneity is balanced (27). Leave-one-out analysis was also performed to test whether a single SNP biased the MR estimate. Additionally, we also employed the MR Steiger directionality test to examine whether the directionality from body fat distribution to AAM is accurate.

In the MR-BMA's analysis, we used the *Q*-statistic to quantify the outlier and the Cook's distance to quantify the influential variants (19). Any outliers or influential points of genetic variation was removed and reanalyzed.

### 2.7 Power calculation

In the univariable MR analyses, we calculated the statistic power using the method described by Brion et al. (28). (https://shiny.cnsgenomics.com/mRnd/). The equations use an approximate linear model, which requires the proportion of variation in the exposure variable explained by IVs (*R*^2^), the effect size of the exposure to the outcome, sample size, and the variance (*σ*^2^) of the exposure and outcome. A sufficient power of over 80% was recommended.

## 3 Results

### 3.1 Study overview

The current study appraised the causal effect of 8 phenotypes related to fat distribution on AAM. Following stringent procedures for filtering SNPs, the number of SNPs finally used for each of the phenotypes varied from 82 to 105. The *F*-statistics ranged from 29.74 to 225.88, suggesting bias owing to the employment of weak instruments unlikely. Statistical power was presented in Supplementary Table S1.

### 3.2 Causal effect from body fat distribution to AAM

The results of the MR analysis were presented in Table 1. In the primary analysis, the causal relationships between all eight exposures and the outcome were identified at a significance level (*P* < 0.05). After Bonferroni correction (*P* < 0.006), statistically significant negative correlations were observed between AAM and whole body fat mass (*β*: −0.17; 95% CI: −0.24, −0.11), left leg fat percentage (*β*: −0.14; 95% CI: −0.21, −0.07), left leg fat mass (*β*: −0.20; 95% CI: −0.27, −0.12), left arm fat percentage (*β*: −0.18; 95% CI: −0.26, −0.11), left arm fat mass (*β*: −0.18; 95% CI: −0.26, −0.10). And the beta coefficients for each of the three MR evaluation methods exhibited consistent trends.

**Table 1:** Mendelian randomization estimates for the associations between body fat distribution and age at menarche.

Exposure	Estimates method	SNPs, N	*β* (95% CI)	*P* value
Body fat percentage	IVW	89	−0.09 (−0.17, −0.01)	0.04*
	MR Egger	89	−0.01 (−0.40, 0.38)	0.96
	Weighted median	89	−0.04 (−0.12, 0.03)	0.26
Whole body fat mass	IVW	105	−0.17 (−0.24, −0.11)	5.06 × 10^−7^**
	MR Egger	105	−0.46 (−0.77, −0.14)	0.01*
	Weighted median	105	−0.18 (−0.25, −0.11)	2.76 × 10^−7^**
Left leg fat percentage	IVW	92	−0.14 (−0.21, −0.07)	1.57 × 10^−4^**
	MR Egger	92	−0.29 (−0.66, 0.09)	0.14
	Weighted median	92	−0.12 (−0.20, −0.05)	1.34 × 10^−3^**
Left leg fat mass	IVW	95	−0.20 (−0.27, −0.12)	3.32 × 10^−7^**
	MR Egger	95	−0.28 (−0.57, 0.01)	0.07
	Weighted median	95	−0.18 (−0.25, −0.11)	1.48 × 10^−6^**
Left arm fat percentage	IVW	90	−0.18 (−0.26, −0.11)	3.04 × 10^−6^**
	MR Egger	90	−0.15 (−0.51, 0.22)	0.44
	Weighted median	90	−0.19 (−0.27, −0.11)	3.08 × 10^−6^**
Left arm fat mass	IVW	91	−0.18 (−0.26, −0.10)	6.29 × 10^−6^**
	MR Egger	91	−0.18 (−0.49, 0.14)	0.27
	Weighted median	91	−0.17 (−0.24, −0.09)	1.35 × 10^−5^**
Trunk fat percentage	IVW	82	0.09 (0.01, 0.16)	0.03*
	MR Egger	82	0.18 (−0.12, 0.48)	0.25
	Weighted median	82	0.01 (−0.07, 0.08)	0.91
Trunk fat mass	IVW	103	−0.10 (−0.17, −0.02)	0.01*
	MR Egger	103	0.10 (−0.17, 0.36)	0.47
	Weighted median	103	−0.02 (−0.09, 0.05)	0.51

IVW, inverse variance weighted.

*P < 0.05, **P < 0.006 (0.05 divided by 8 exposures).

Among three nominal significant results, it was observed that body fat percentage (*β*: −0.09; 95% CI: −0.17, −0.01) and trunk fat mass (*β*: −0.10; 95% CI: −0.17, −0.02) exhibited an inverse relationship with AAM, while trunk fat percentage (*β*: 0.09; 95% CI: 0.01, 0.16) showed a positive association with AAM.

### 3.3 Results for sensitivity analyses of univariable MR analyses

In sensitivity analysis, we found that all eight causal relationships exhibit heterogeneity with *I*^2 ^> 50% (Table 2). However, we addressed this issue by applying the IVW method with the random-effect model. The results of the MR Egger intercept test demonstrated that there was no directional pleiotropy observed (Table 2). Additionally, no single SNP strongly violated the results of all causal estimates in the leave-one-out analysis (Supplementary Figure S1). The analysis results of the scatter plots were shown in Supplementary Figure S2. Furthermore, the results of the MR Steiger directionality test confirmed that the direction of our evaluated causal relationship was accurate (*P* < 0.001).

**Table 2:** Heterogeneity and pleiotropy assessment for inverse variance weighted method in univariable MR analyses.

Exposure	*β* (95% CI)	Cochran's *Q*-derived *P* value	*I* ^2^	MR-egger intercept derived *P* value
Body fat percentage	−0.09 (−0.17, −0.01)	3.22 × 10^−31^	74.08%	0.68
Whole body fat mass	−0.17 (−0.24, −0.11)	2.80 × 10^−21^	65.63%	0.08
Left leg fat percentage	−0.14 (−0.21, −0.07)	9.71 × 10^−15^	61.34%	0.43
Left leg fat mass	−0.20 (−0.27, −0.12)	1.84 × 10^−27^	71.23%	0.58
Left arm fat percentage	−0.18 (−0.26, −0.11)	1.59 × 10^−22^	68.89%	0.83
Left arm fat mass	−0.18 (−0.26, −0.10)	2.57 × 10^−29^	72.83%	0.96
Trunk fat percentage	0.09 (0.01, 0.16)	5.58 × 10^−16^	64.54%	0.53
Trunk fat mass	−0.10 (−0.17, −0.02)	2.11 × 10^−34^	73.48%	0.14

### 3.4 Results of MR Bayesian model averaging (MR-BMA) analysis

We further performed a MR-BMA analysis with five body fat distribution traits that were identified in the univariable MR analyses on AAM. A total of 338 SNPs were found to be associated with these traits after removing duplicate SNPs. Subsequently, we excluded 144 SNPs that in LD (*r*^2 ^> 0.001), 26 SNPs that were unavailable in the outcome dataset, and 10 SNPs that were outliers based on the *Q*-statistic for further analysis. No influential genetic variants were identified in the analysis based on Cook's distance.

In the MR-BMA analysis, the top ten models with the highest posterior probability were presented in Supplementary Table S2. After that, the MIPs of all five exposures were calculated and used to rank these exposures for their causal associations with AAM (Table 3). Left arm fat percentage was identified as the highest-ranked trait exhibiting a negative causal association with AAM (MIP = 0.317, average effect = −0.048, *P* = 0.042), followed by left leg fat percentage (MIP = 0.305, average effect = −0.045, *P* = 0.029).

**Table 3:** Ranking of five body fat distribution traits for the risk of age at menarche using MR-BMA method.

Exposure	Ranking by MIP	MIP	Average effect	*P* value
Left arm fat percentage	1	0.317	−0.048	0.042
Left leg fat percentage	2	0.305	−0.045	0.028
Left leg fat mass	3	0.183	−0.026	0.984
Whole body fat mass	4	0.175	−0.023	0.991
Left arm fat mass	5	0.088	−0.004	0.999

MIP, marginal inclusion probability.

## 4 Discussion

To our knowledge, this is the first MR study to investigate the correlation of a series of indicators about body fat distribution and AAM. In our two-sample MR study, we discovered that five indicators (whole body fat mass, left leg fat percentage, left leg fat mass, left arm fat percentage, and left arm fat mass) pertaining to body fat distribution demonstrated a negative causal correction with the AAM (*P* < 0.006). This suggests that as these indicators increase, the age of girls experiencing their first menarche decreases. Among the aforementioned variables, the most significant influence on AAM was observed in the left arm fat percentage based on the MR-BMA analysis. These findings underscore the significance of incorporating body fat distribution indicators, in addition to the conventional body mass index (BMI), when investigating the association between obesity and pubertal development.

In previous observational studies conducted in Brazil, Chile and the United States, researchers also discovered a correlation between fat mass and the AAM (13, 14), and girls with higher total body fat (TBF) achieved menarche earlier than girls with lower TBF (15). This was consistent with our findings. However, in the study conducted by Wang et al. (16) in the Chinese population, they found that girls with precocious puberty had a higher trunk fat percentage than the normal controls, which contradicted the findings of our study. Our study, on the other hand, revealed a positive association between trunk fat percentage and AAM, although statistical significance was not achieved following adjustments for multiple comparisons (0.006 < *P* < 0.05). This could be due to the different race populations and heterogeneous measurement of fat percentage. And in the study by Wang et al., the diagnosis of precocious puberty was not solely dependent on the AAM.

The mechanisms of body fat can influence the onset of menarche mainly due to the impact of excessive adipose tissue on the body's endocrine system and levels of sex hormones. An increase in body fat content can lead to elevated levels of estrogen in the body, disrupting the normal balance of sex hormones and potentially triggering early menarche (29). In addition, individuals who are overweight or obese often experience insulin resistance and high levels of insulin. This can result in reduced concentrations of sex hormone-binding globulin. Thus, the bioavailability of sex steroids is potentially increased, which can alter the timing and progression of puberty (30). Furthermore, an increase in body fat mass may serve as a significant signal for triggering leptin secretion. Leptin secretion stimulates the hypothalamus, leading to the excessive release of gonadotropin-releasing hormone (GnRH), thereby stimulating the hypothalamus-pituitary-gonad (PHG) axis, and initiating pubertal development (31).

Our work has several important strengths. We employed an MR framework to investigate the causal relationship between body fat distribution and AAM, we also ranked the body fat distribution indicators for their causal associations by MR-BMA analysis. By utilizing genetic variants as instrumental variants, MR design allows us to minimize the influence of potential confounding factors, such as lifestyle factors like physical activity and dietary patterns, which are often present in traditional observational studies. Furthermore, by exclusively utilizing datasets that primarily consist of individuals of European descent, the potential influence of population stratification on the observed results was minimized. Finally, the exposed data we utilized only included data from females, which corresponds to the outcome variable. Several limitations should be considered in our study. First, the AAM of the subjects was determined through self-report and recall, which may introduce bias into the data. Second, the outcome data were derived from a meta-GWAS, and each included study may have different confounders for analysis, leading to high heterogeneity and potential bias.

In conclusion, our research has revealed a causal relationship between several indicators of body fat distribution and the AAM, and identified that left arm fat percentage was the most influential factor. These findings will be particularly useful for identifying girls who might experience earlier menarche. Furthermore, these results possess the potential to enhance the accuracy and robustness of AAM prediction models, rendering them invaluable in practice. In the future, obtaining more information on body measurements during the prepubertal stage would greatly enhance the precision of our comprehension in this domain.

## Data Availability

Publicly available datasets were analyzed in this study. This data can be found here: the website of UK Biobank (http://www.nealelab.is/uk-biobank) and ReproGen consortium (https://www.reprogen.org).
