# Supplementary material for: Association between body fat distribution and age at menarche: a two sample Mendelian randomization study
Source: Front Pediatr. 2024 Apr 8;12:1349670. doi: 10.3389/fped.2024.1349670 (PMC11033318; doi:10.3389/fped.2024.1349670)
Supplement: Supplementary file 1 [file Table1.docx]

**Table S1. Statistical power calculation for Mendelian randomization effects derived from random-effects IVW method.**

| **Exposure** | **SNPs, N** | **R^2^** | ***F*-statistics** | **Power** |
| --- | --- | --- | --- | --- |
| Body fat percentage | 89 | 0.02 | 29.80-110.74 | 100% |
| Whole body fat mass | 105 | 0.02 | 29.75-88.91 | 100% |
| Left leg fat percentage | 92 | 0.02 | 29.74-108.50 | 100% |
| Left leg fat mass | 95 | 0.02 | 29.82-225.88 | 100% |
| Left arm fat percentage | 90 | 0.02 | 29.90-134.67 | 100% |
| Left arm fat mass | 91 | 0.02 | 29.96-217.06 | 99% |
| Trunk fat percentage | 82 | 0.02 | 30.09-119.89 | 100% |
| Trunk fat mass | 103 | 0.03 | 29.87-180.71 | 100% |

IVW: Inverse variance weighted.

R^2^ of single nucleotide polymorphism (SNP) was calculated by using the formula R^2^ = [2 × 𝐵𝐸𝑇𝐴^2^ × 𝐸𝐴𝐹 × (1 − 𝐸𝐴𝐹)] / [2 × 𝐵𝐸𝑇𝐴^2^ × 𝐸𝐴𝐹 × (1 − 𝐸𝐴𝐹) + 2 × *SE*(𝐵𝐸𝑇𝐴)^2^ × 𝑁 × 𝐸𝐴𝐹 × (1 − 𝐸𝐴𝐹)]. The cumulative R^2^ of each individual SNP under that exposure phenotype was subsequently computed to represent the variability that these SNPs explained. Here, BETA is the genetic effects on exposures; EAF is effect allele frequency; SE(BETA) is standard error of the genetic effects; N is the sample size. *F*-statistic quantified the strength of each selected SNP using the formula 𝑅^2^ × (𝑁 −2) / (1 −𝑅^2^).

**Table S2. Top ten best models and corresponding posterior probabilities in MR-BMA analysis.**

| **Combination** | **Posterior probability** | **Causal estimate** |
| --- | --- | --- |
| Left arm fat percentage | 0.295 | -0.155 |
| Left leg fat percentage | 0.278 | -0.147 |
| Left leg fat mass | 0.154 | -0.140 |
| Whole body fat mass | 0.147 | -0.139 |
| Left arm fat mass | 0.061 | -0.132 |
| Left leg fat mass, Left arm fat mass | 0.009 | -0.363, 0.219 |
| Left leg fat percentage, Left arm fat percentage | 0.007 | -0.074, -0.079 |
| Whole body fat mass, Left leg fat percentage | 0.007 | 0.046, -0.194 |
| Whole body fat mass, Left leg fat mass | 0.007 | -0.043, -0.097 |
| Whole body fat mass, Left arm fat mass | 0.007 | -0.334, 0.194 |
