# Supplementary figures and images for: Association between body fat distribution and age at menarche: a two sample Mendelian randomization study
Source: Front Pediatr. 2024 Apr 8;12:1349670. doi: 10.3389/fped.2024.1349670 (PMC11033318; doi:10.3389/fped.2024.1349670)

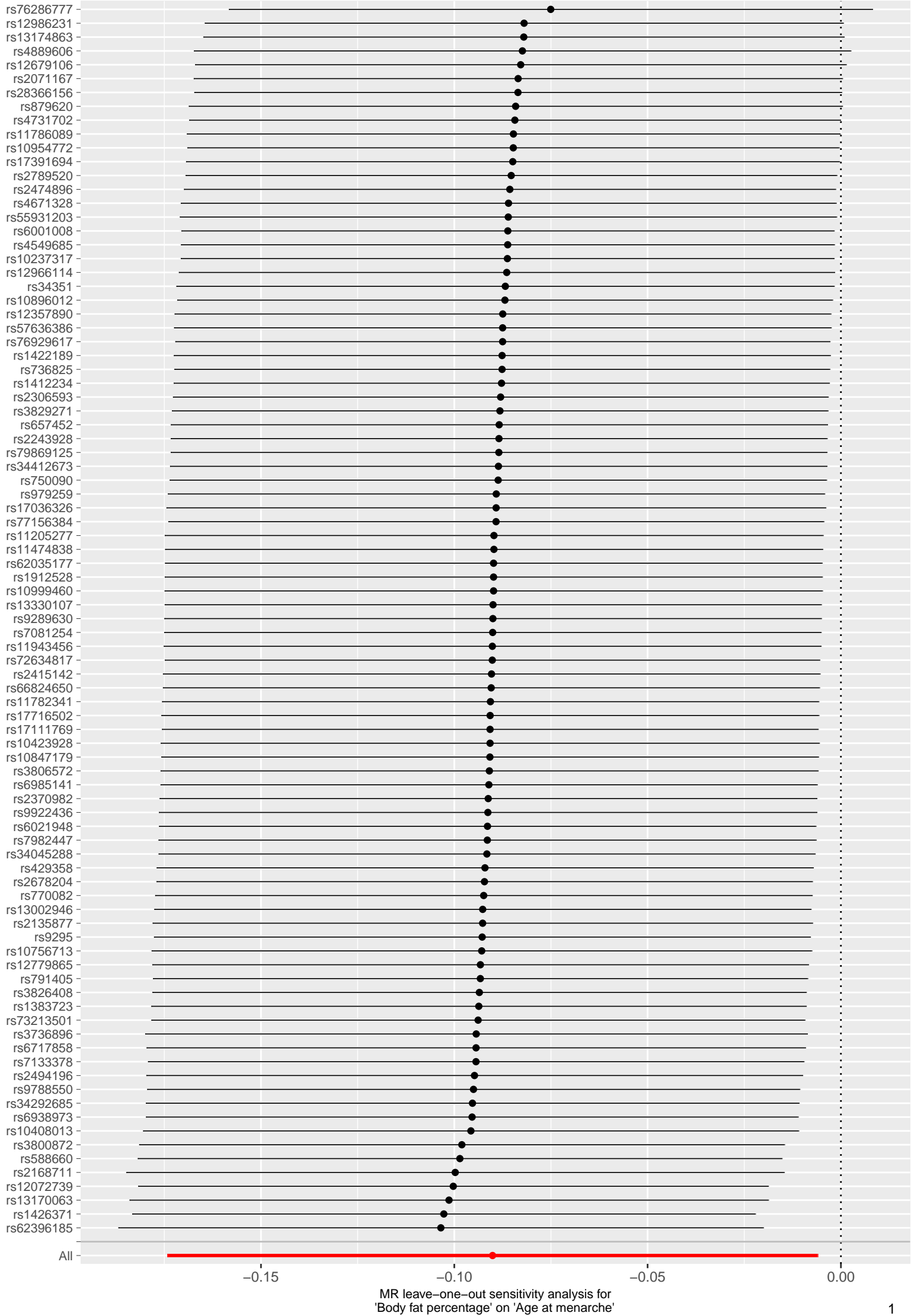

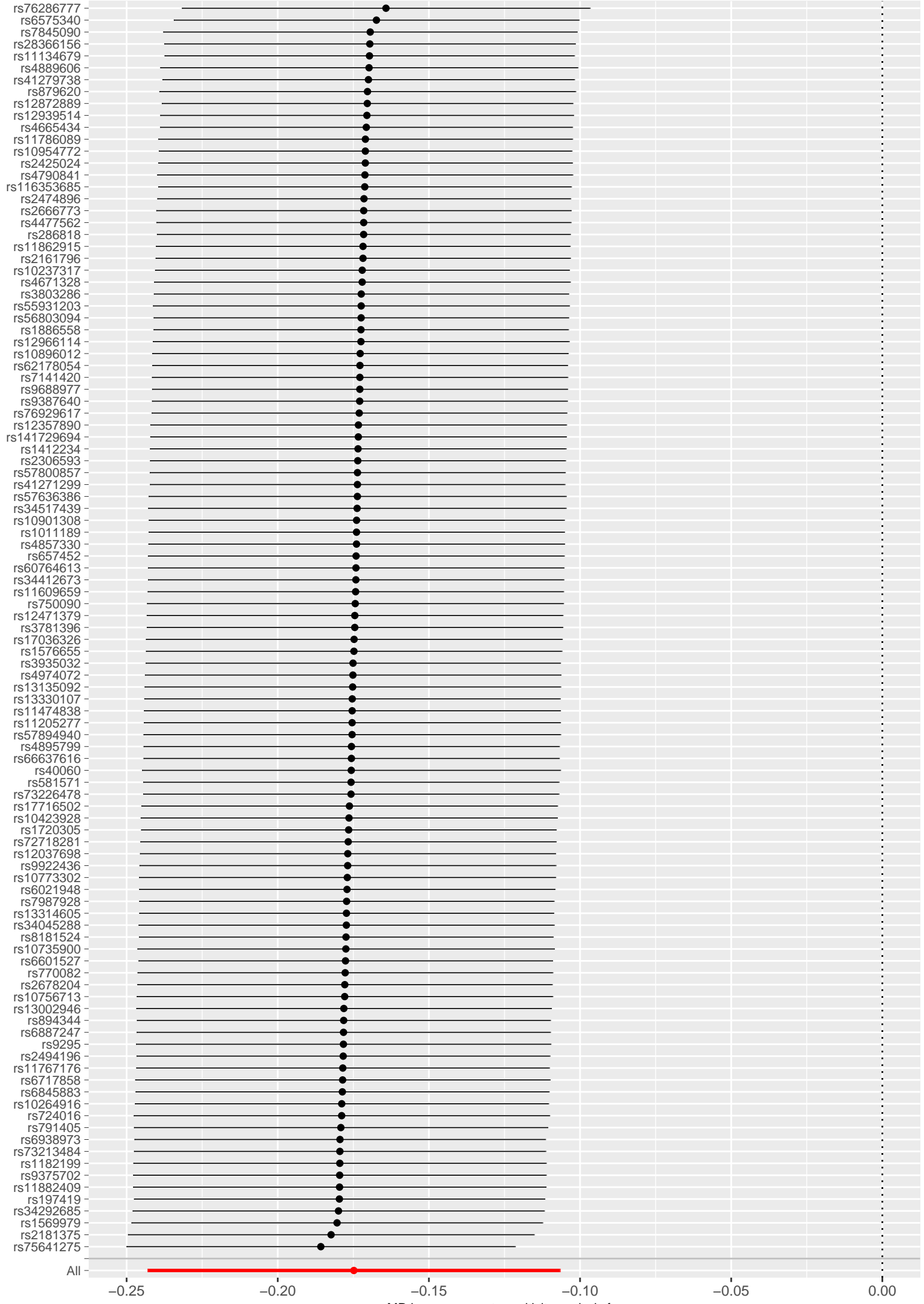

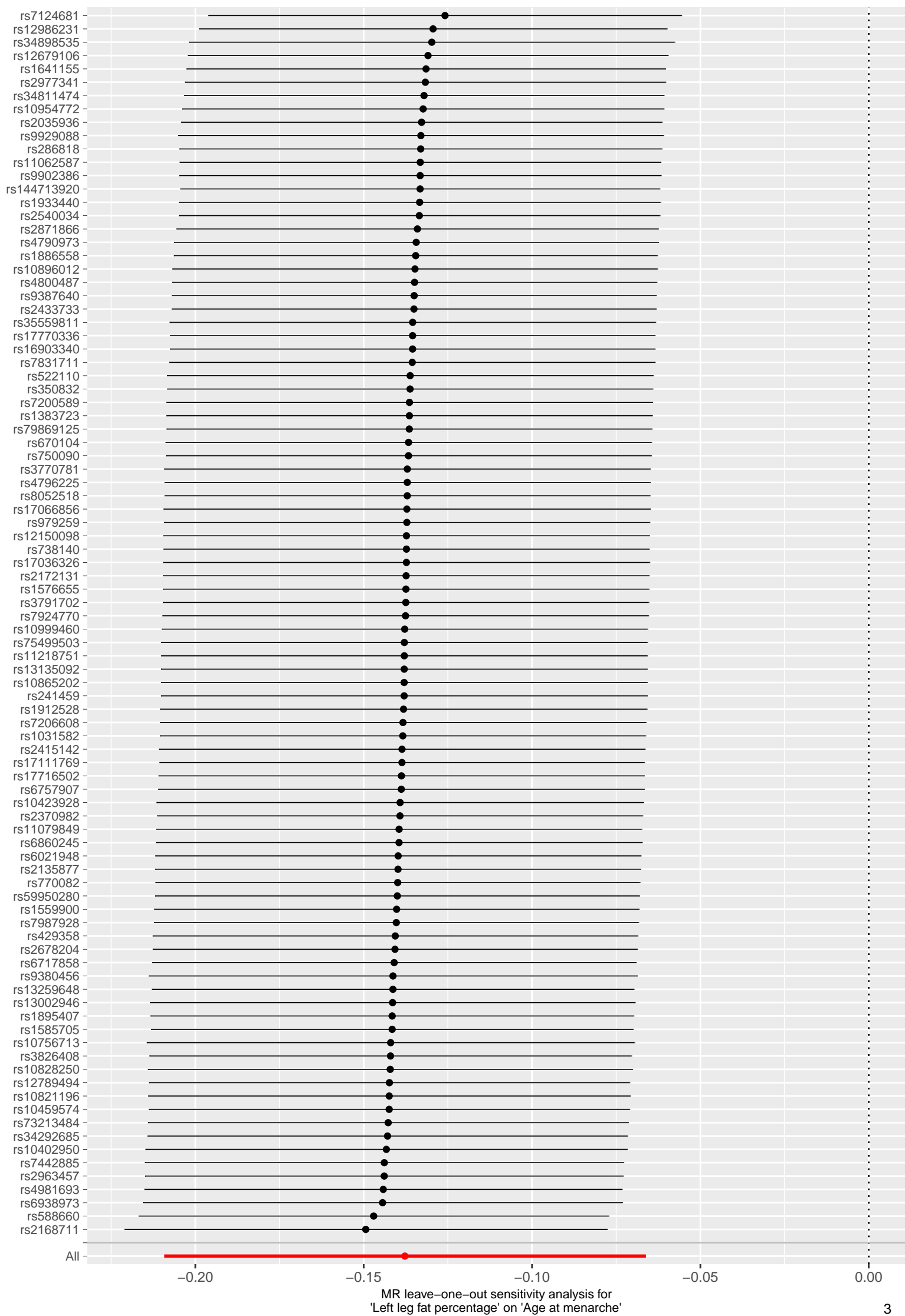

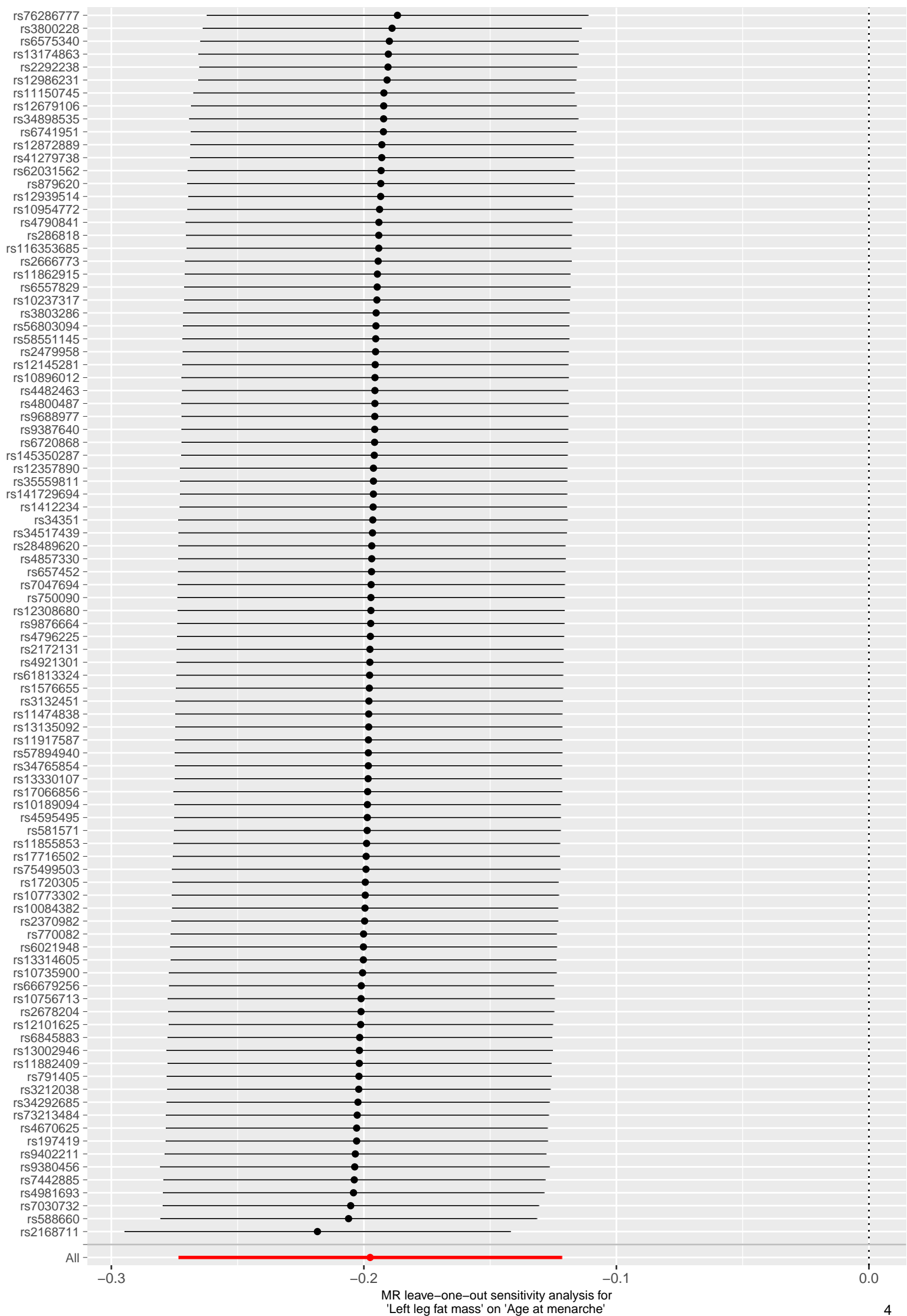

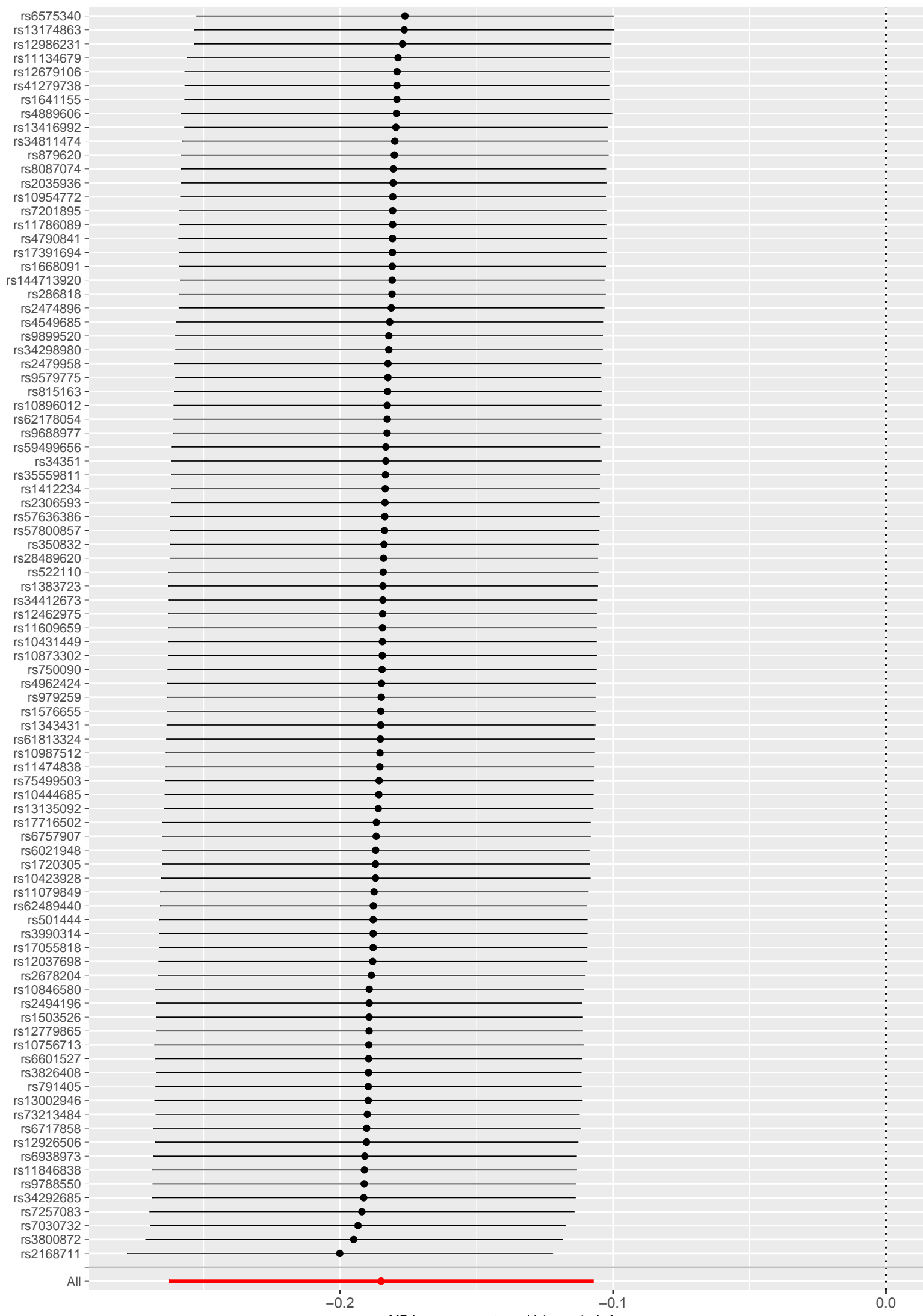

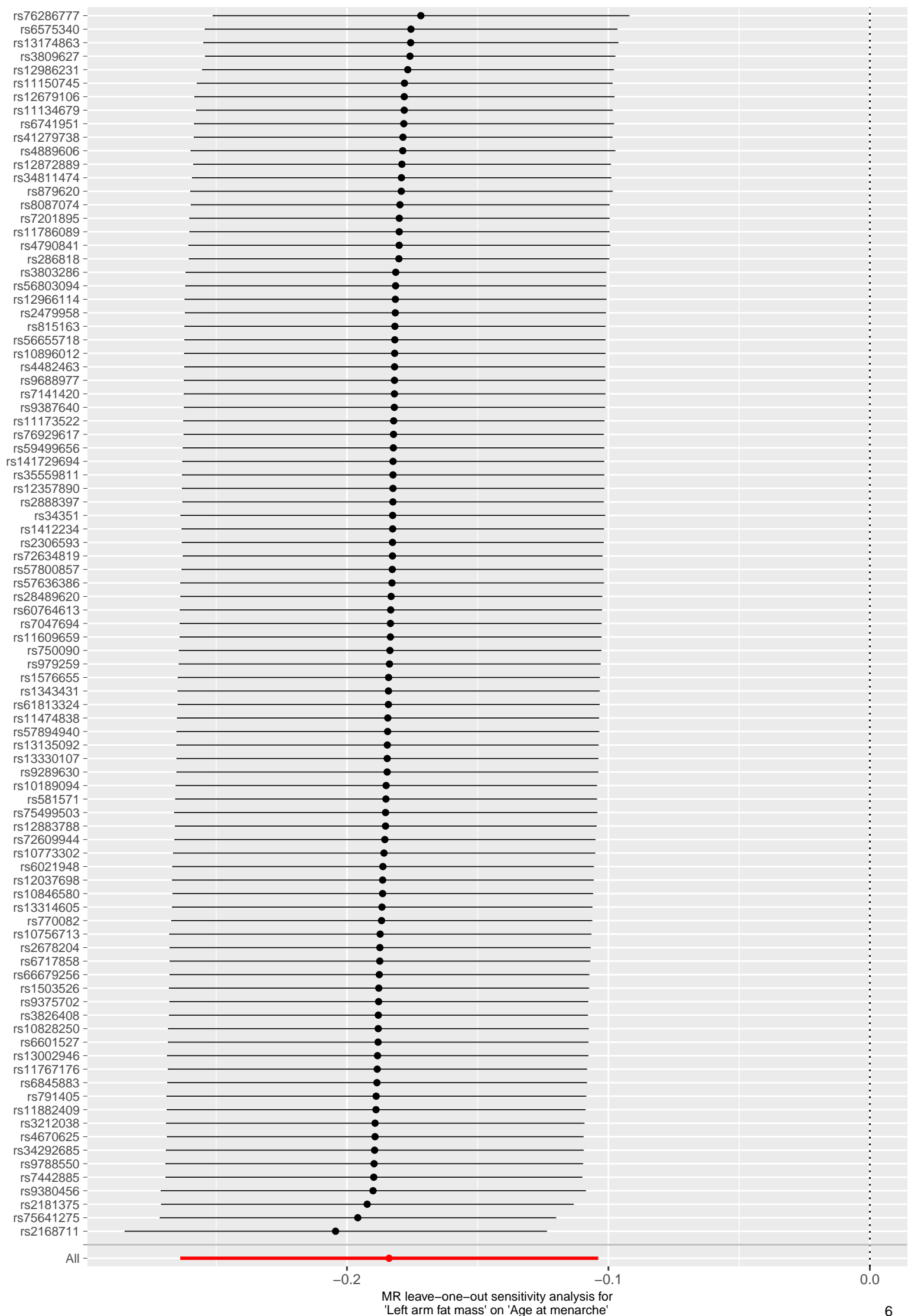

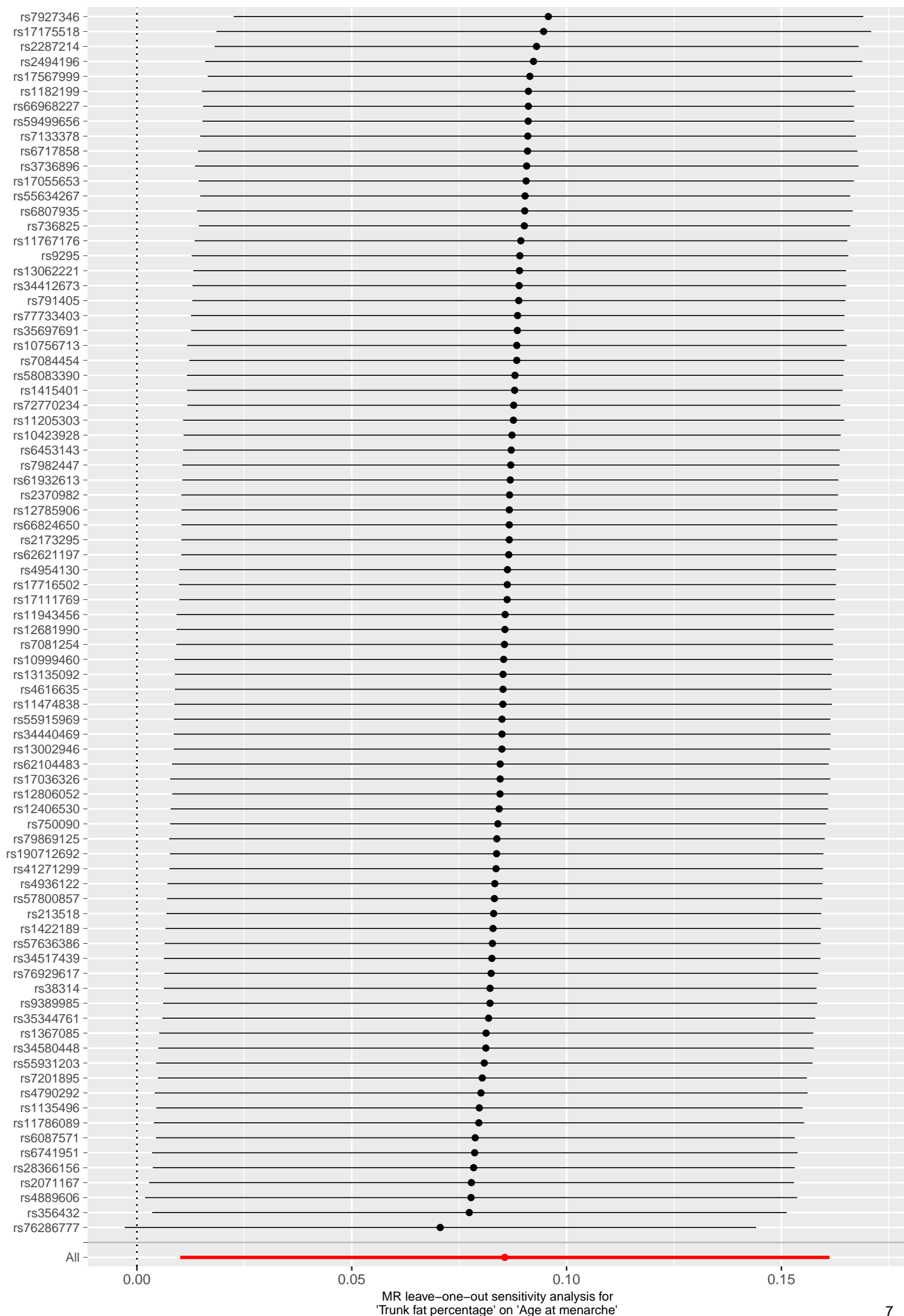

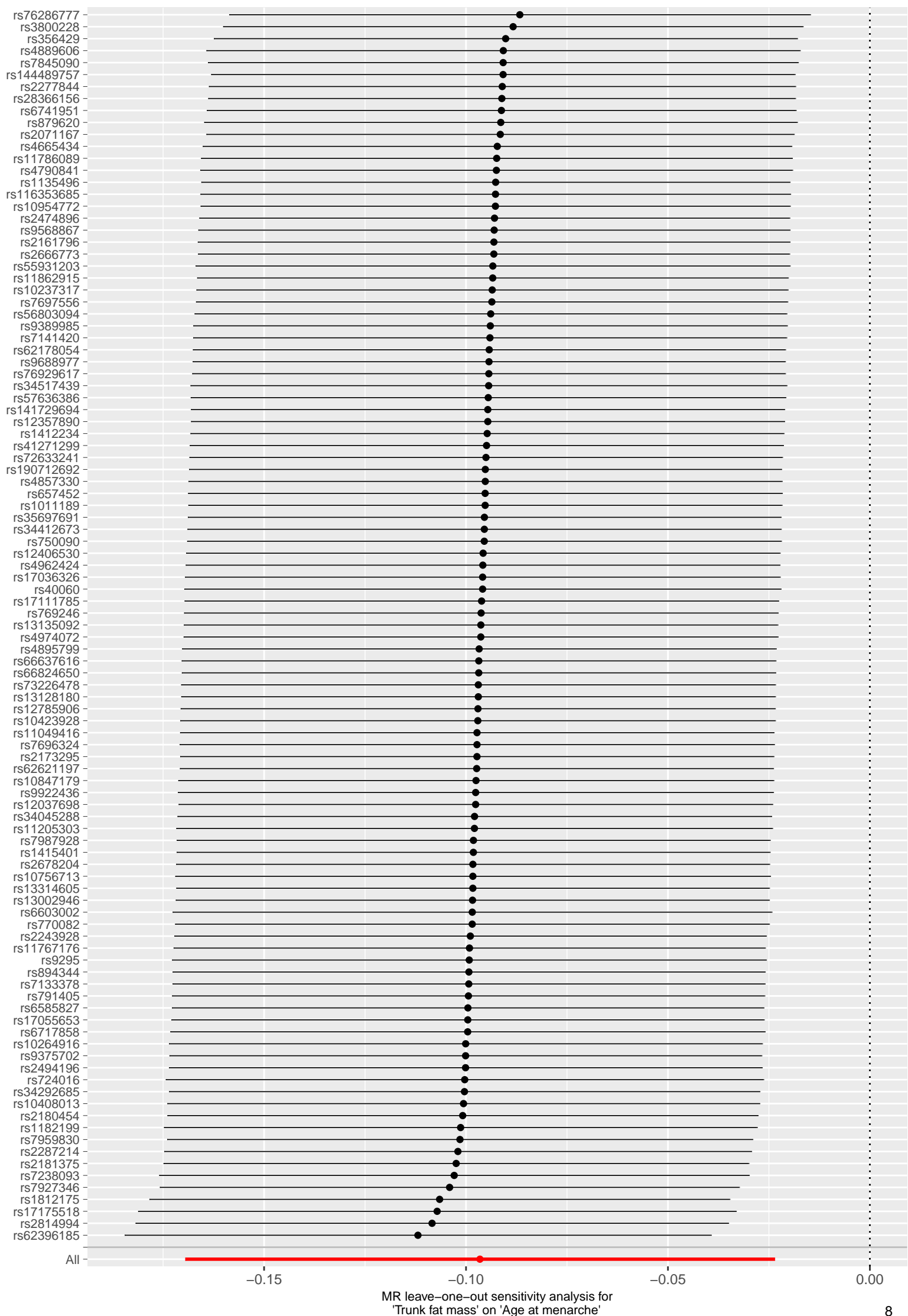

Supplement: Supplementary file 2 [file Image1.pdf]
