# Supplementary material for: Association between body fat distribution and age at menarche: a two sample Mendelian randomization study
Source: Front Pediatr. 2024 Apr 8;12:1349670. doi: 10.3389/fped.2024.1349670 (PMC11033318; doi:10.3389/fped.2024.1349670)
Supplement: Supplementary file 3 [file Image2.pdf]

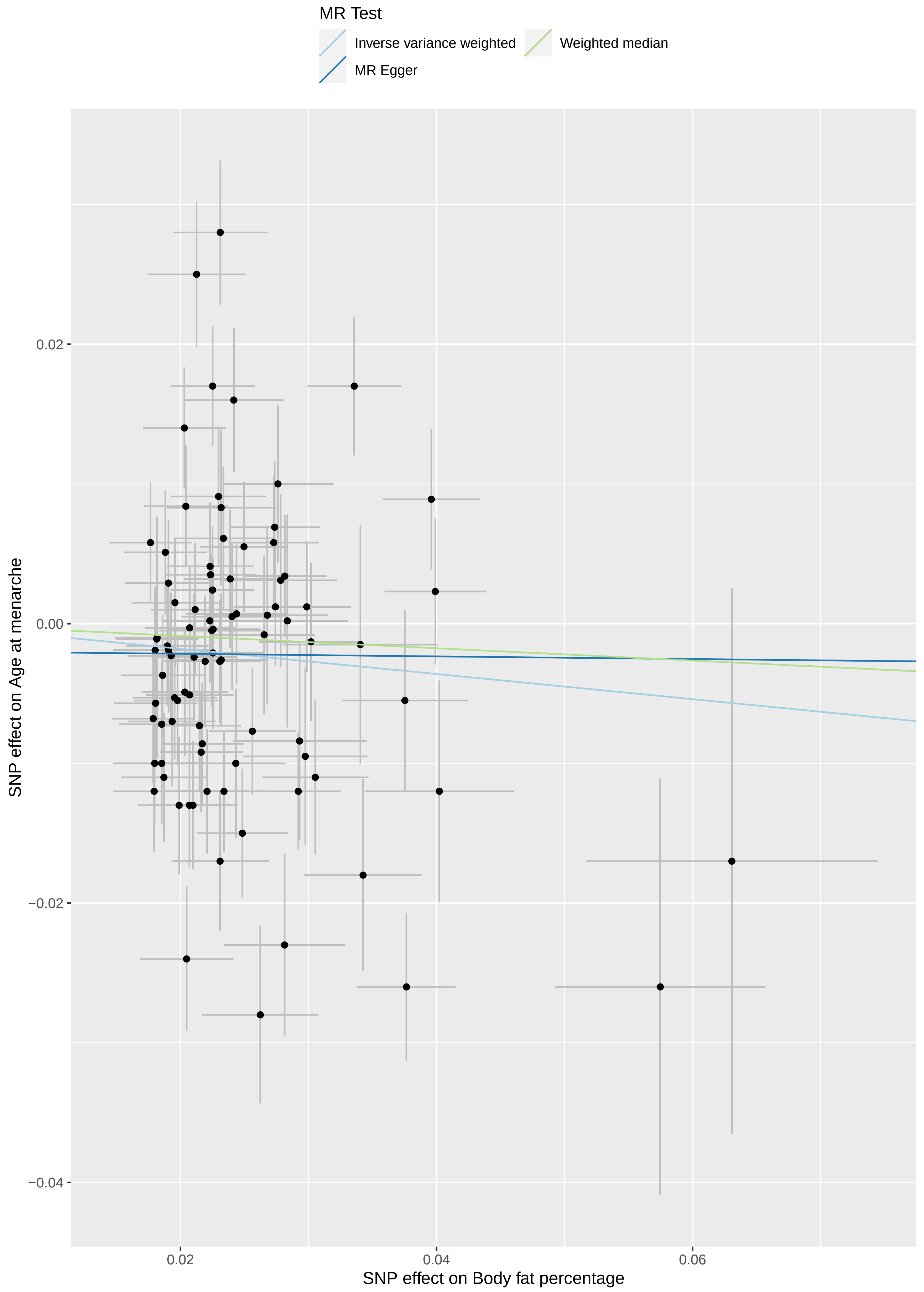
MR Test
Inverse variance weighted
Weighted median
MR Egger
SNP effect on Age at menarche
0.02
0.00
−0.02
−0.04
0.02
0.04
0.06
SNP effect on Body fat percentage

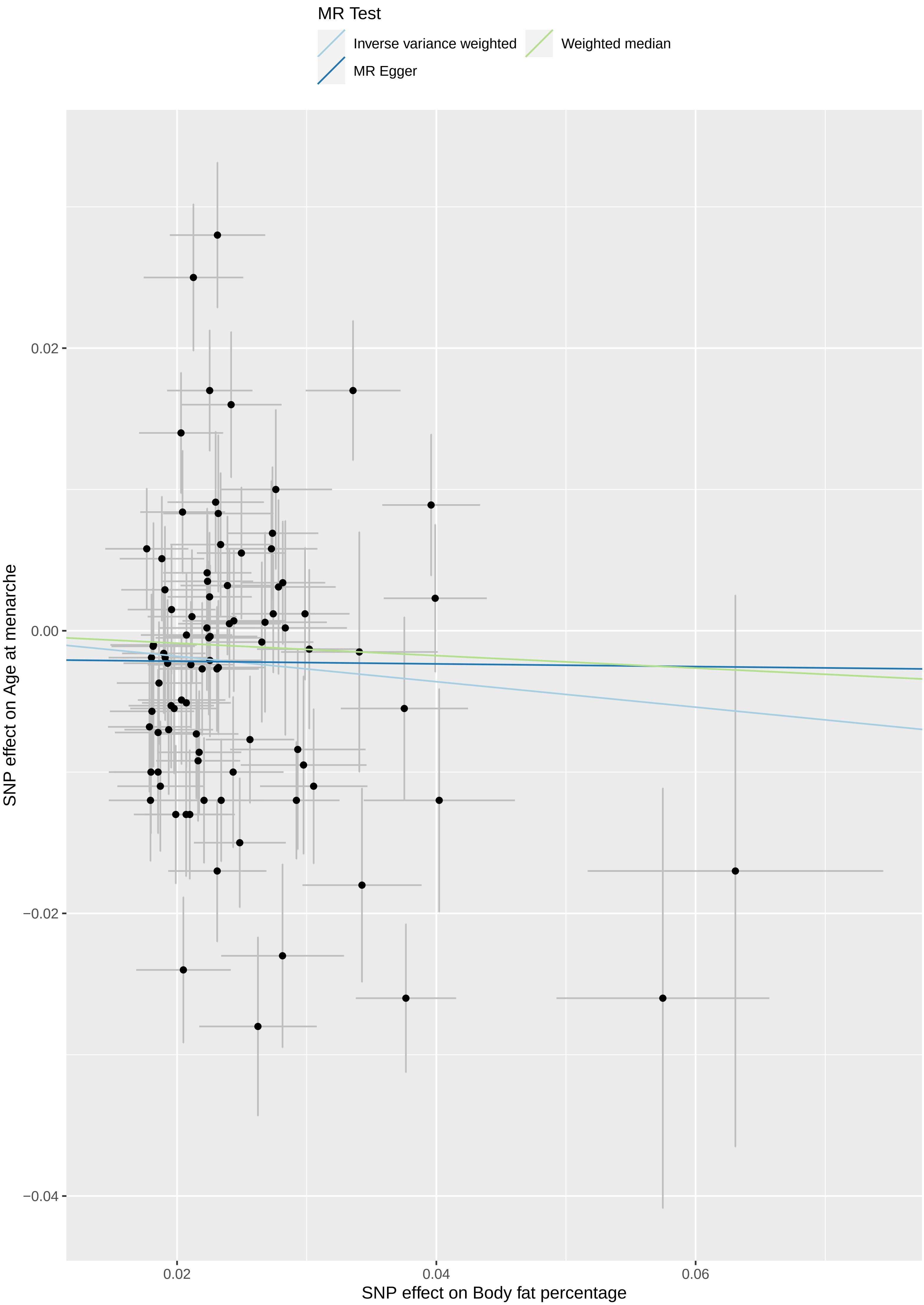
MR Test
Inverse variance weighted
Weighted median
MR Egger
0.02
0.00
−0.02
−0.04
0.02
0.04
0.06
SNP effect on Age at menarche
SNP effect on Body fat percentage

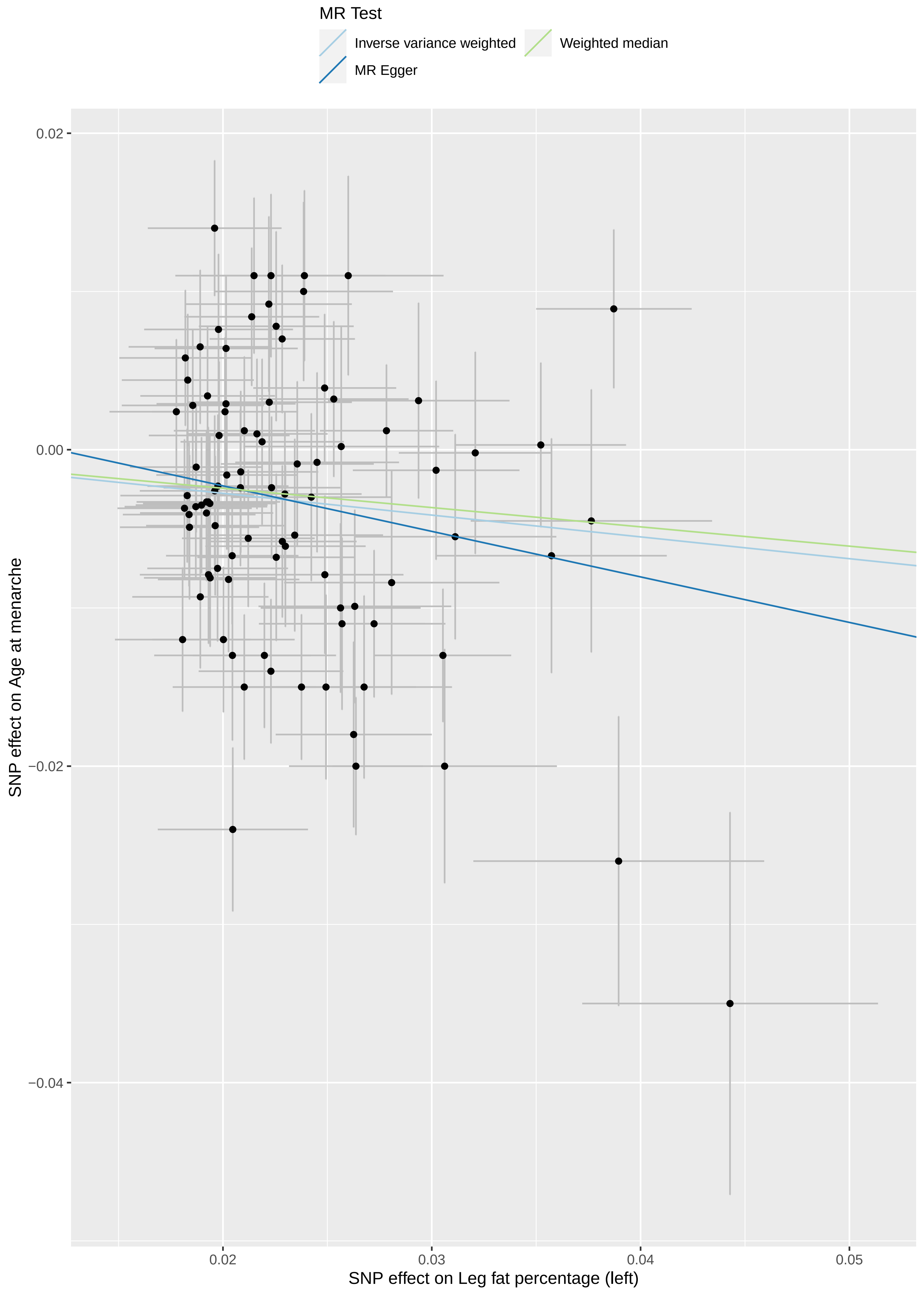
MR Test
Inverse variance weighted
Weighted median
MR Egger
0.02
0.00
−0.02
−0.04
SNP effect on Age at menarche
0.02
0.03
0.04
0.05
SNP effect on Leg fat percentage (left)

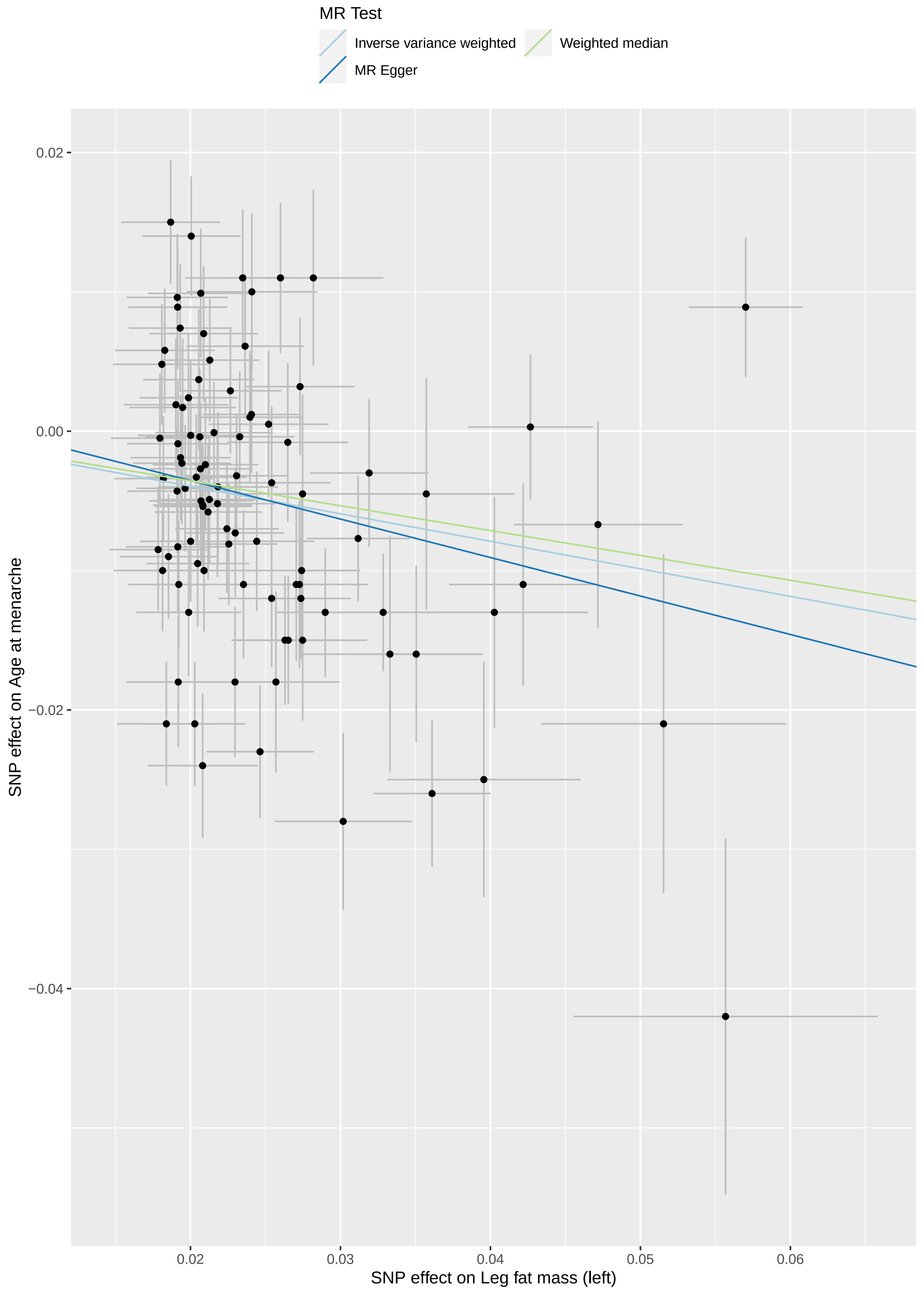

MR Test
Inverse variance weighted
MR Egger
Weighted median
0.02
0.00
−0.02
−0.04
SNP effect on Age at menarche
0.02
0.03
0.04
0.05
0.06
SNP effect on Leg fat mass (left)

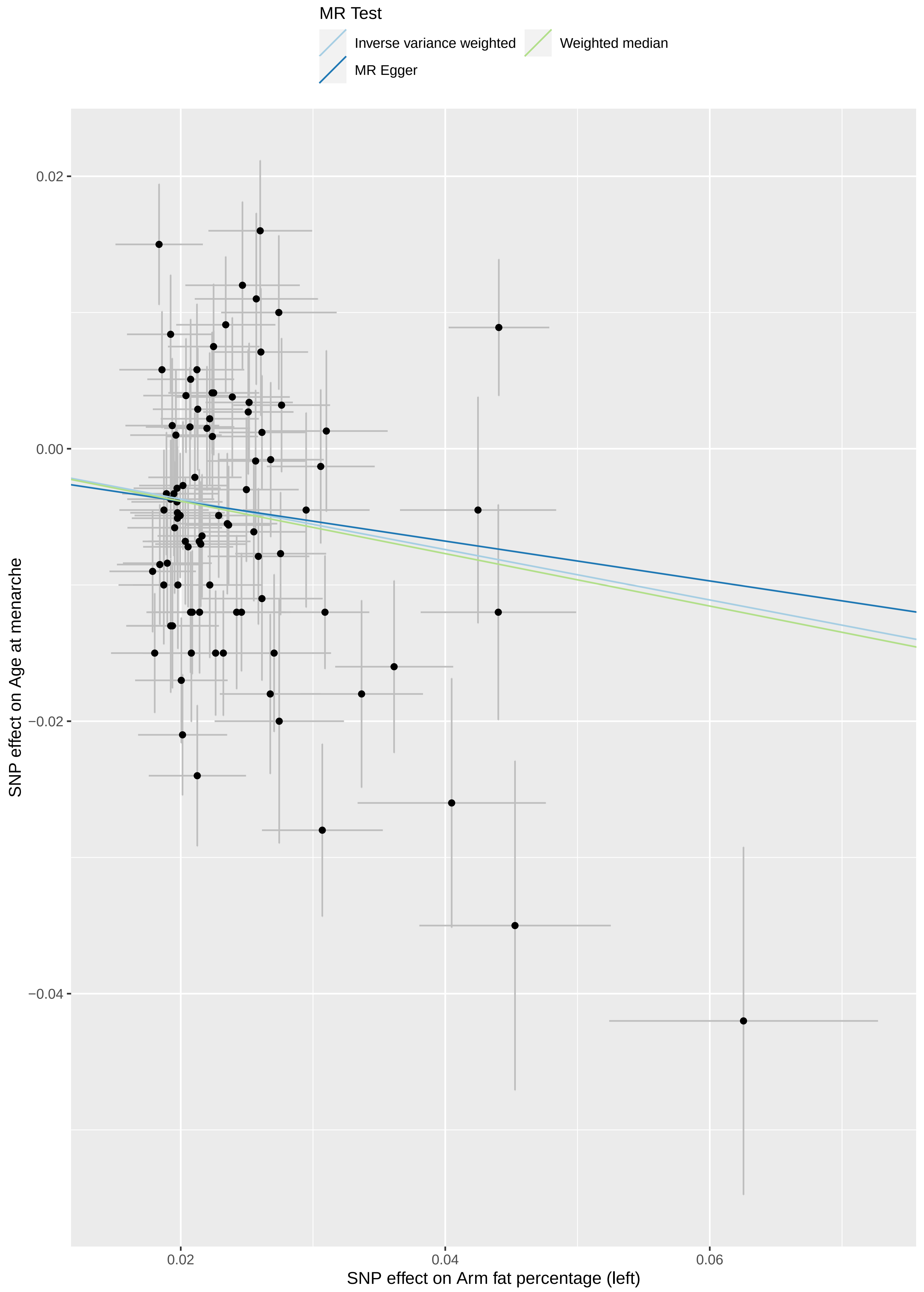

MR Test
Inverse variance weighted
Weighted median
MR Egger
0.02
0.00
−0.02
−0.04
SNP effect on Age at menarche
0.02
0.04
0.06
SNP effect on Arm fat percentage (left)

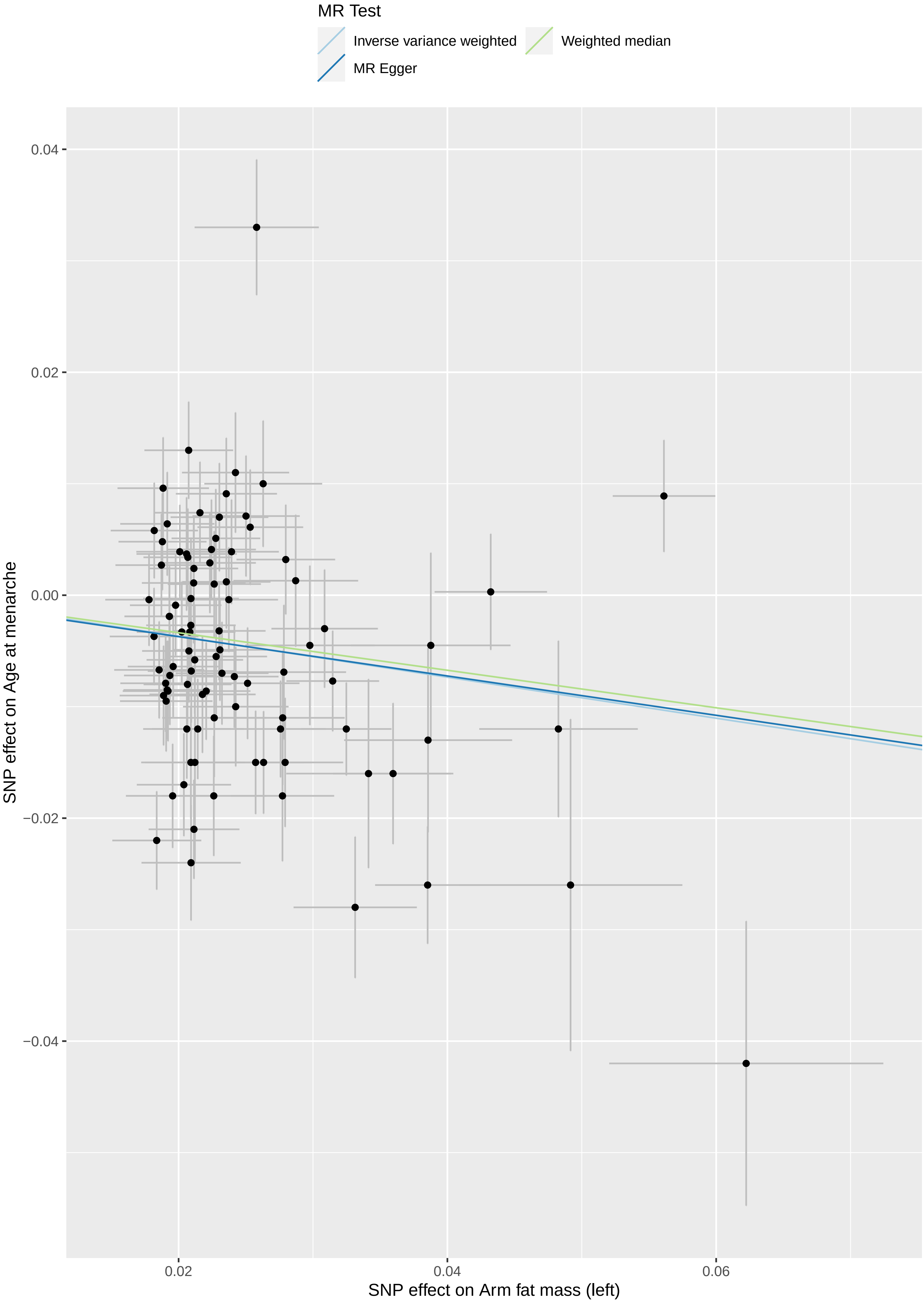
MR Test
Inverse variance weighted
Weighted median
MR Egger
0.04
0.02
0.00
−0.02
−0.04
SNP effect on Age at menarche
0.02
0.04
0.06
SNP effect on Arm fat mass (left)

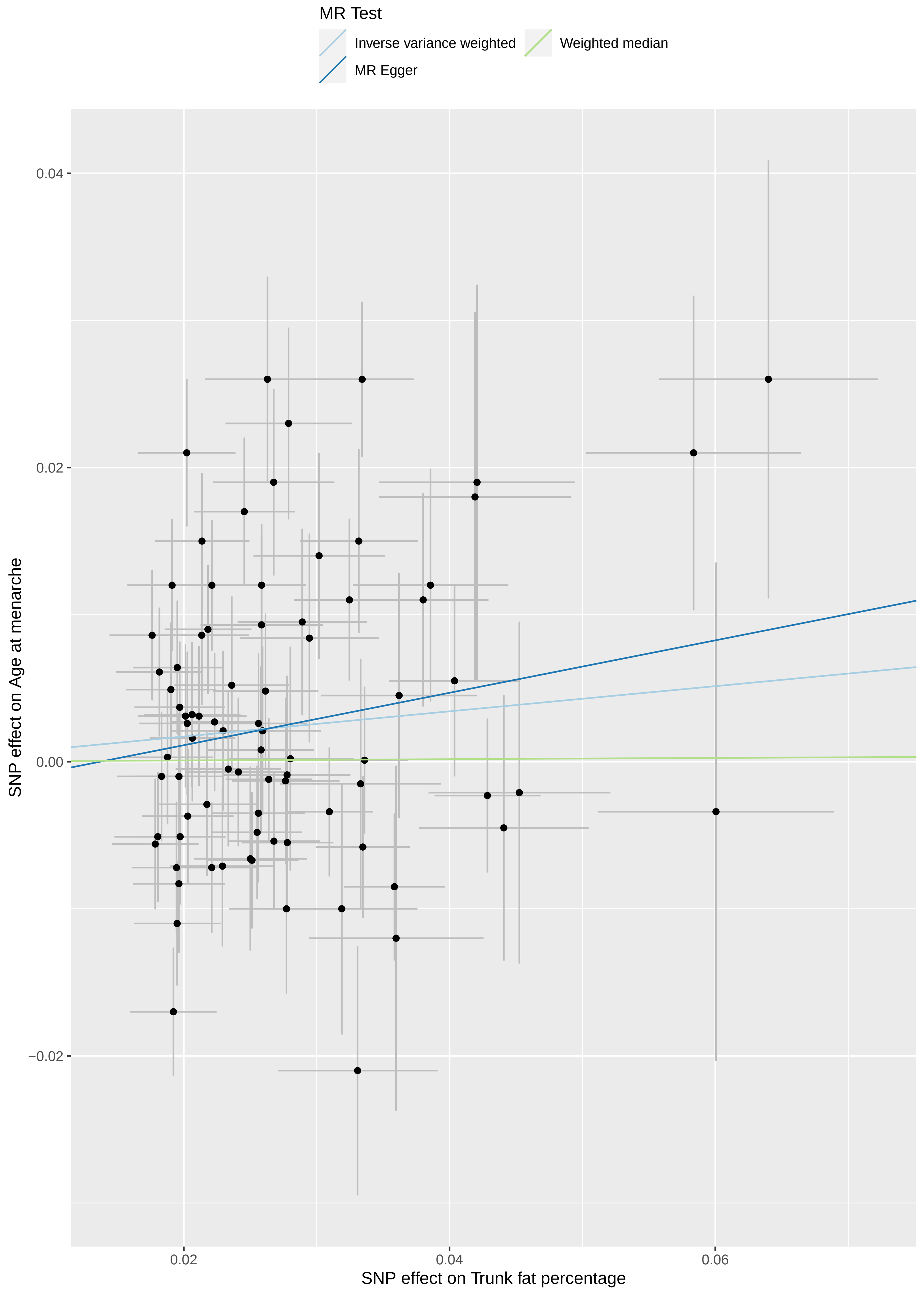
MR Test
Inverse variance weighted
Weighted median
MR Egger
SNP effect on Age at menarche
0.04
0.02
0.00
−0.02
0.02
0.04
0.06
SNP effect on Trunk fat percentage

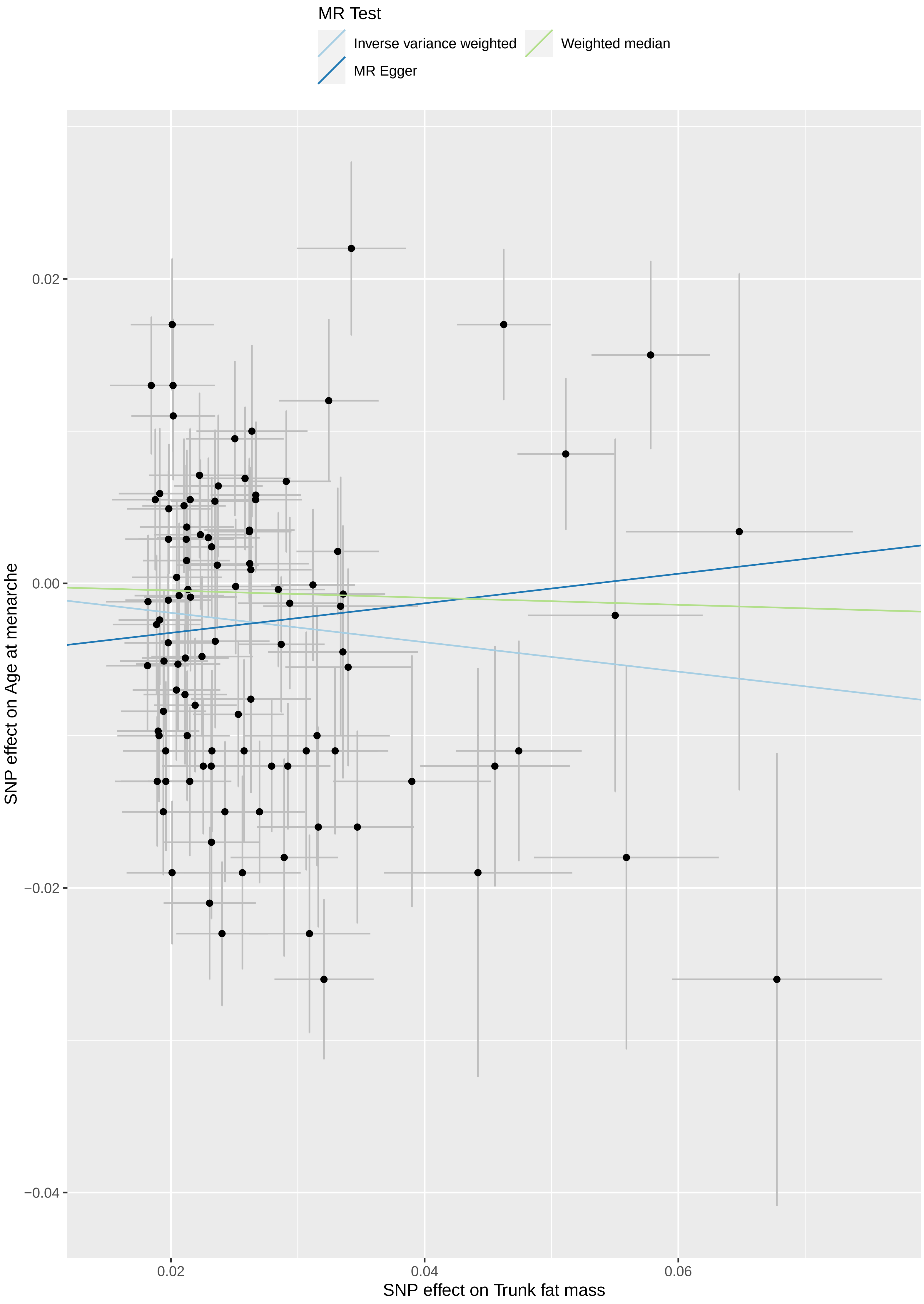
MR Test
Inverse variance weighted
Weighted median
MR Egger
0.02
0.00
−0.02
−0.04
0.02
0.04
0.06
SNP effect on Trunk fat mass
SNP effect on Age at menarche
